# Unsupervised Machine Learning Algorithms Examine Healthcare Providers' Perceptions and Longitudinal Performance in a Digital Neonatal Resuscitation Simulator

**DOI:** 10.3389/fped.2020.00544

**Published:** 2020-09-11

**Authors:** Chang Lu, Simran K. Ghoman, Maria Cutumisu, Georg M. Schmölzer

**Affiliations:** ^1^Department of Educational Psychology, Faculty of Education, Centre for Research in Applied Measurement and Evaluation, University of Alberta, Edmonton, AB, Canada; ^2^Centre for the Studies of Asphyxia and Resuscitation, Neonatal Research Unit, Royal Alexandra Hospital, Edmonton, AB, Canada; ^3^Department of Pediatrics, Faculty of Medicine and Dentistry, University of Alberta, Edmonton, AB, Canada; ^4^Department of Computing Science, Faculty of Science, University of Alberta, Edmonton, AB, Canada

**Keywords:** education, training, simulation, resuscitation, table-top simulator, serious games, digital simulator, medical education

## Abstract

**Background:** Frequent simulation-based education is recommended to improve health outcomes during neonatal resuscitation but is often inaccessible due to time, resource, and personnel requirements. Digital simulation presents a potential alternative; however, its effectiveness and reception by healthcare professionals (HCPs) remains largely unexplored.

**Objectives:** This study explores HCPs' attitudes toward a digital simulator, technology, and mindset to elucidate their effects on neonatal resuscitation performance in simulation-based assessments.

**Methods:** The study was conducted from April to August 2019 with 2-month (June–October 2019) and 5-month (September 2019–January 2020) follow-up at a tertiary perinatal center in Edmonton, Canada. Of 300 available neonatal HCPs, 50 participated. Participants completed a demographic survey, a pretest, two practice scenarios using the RETAIN neonatal resuscitation digital simulation, a posttest, and an attitudinal survey (100% response rate). Participants repeated the posttest scenario in 2 months (86% response rate) and completed another posttest scenario using a low-fidelity, tabletop simulator (80% response rate) 5 months after the initial study intervention. Participants' survey responses were collected to measure attitudes toward digital simulation and technology. Knowledge was assessed at baseline (pretest), acquisition (posttest), retention (2-month posttest), and transfer (5-month posttest).

**Results:** Fifty neonatal HCPs participated in this study (44 females and 6 males; 27 nurses, 3 nurse practitioners, 14 respiratory therapists, and 6 doctors). Most participants reported technology in medical education as useful and beneficial. Three attitudinal clusters were identified by a hierarchical clustering algorithm based on survey responses. Although participants exhibited diverse attitudinal paths, they all improved neonatal resuscitation performance after using the digital simulator and successfully transferred their knowledge to a new medium.

**Conclusions:** Digital simulation improved HCPs' neonatal resuscitation performance. Medical education may benefit by incorporating technology during simulation training.

## Introduction

It is estimated that annually more than 10% of newborns around the globe require assistance to breathe on their own to make the fetal-to-neonatal transition, mainly due to drastic physiological changes from an intrauterine to extrauterine environment (1, 2). Healthcare professionals (HCPs) are expected to perform a series of complex interventions quickly and precisely, including suction, drying, and respiratory support, to help these newborns make the successful fetal-to-neonatal transition. To meet the high expectations for HCPs on neonatal resuscitation tasks under high-pressure conditions, frequent neonatal training is recommended to help HCPs master and retain knowledge and skills and to minimize human errors in the delivery room (1). However, in traditional medical education, students and HCPs have limited access to hands-on experiences that involve complex and rarely occurring high-risk scenarios, such as neonatal resuscitation (3–5).

The advancement of technology enables medical educators to overcome challenges of traditional medical education, including time constraints, space, and clinical duties. It also helps create teaching opportunities for medical students and HCPs to perform high-stakes, sophisticated medical procedures using computer-based simulations (6–8). Many programs have introduced new media technologies to implement learning and assessment tools, including board games (9, 10), video games (11–15), mobile learning platforms (6, 16–18), virtual environments (19–23), and simulations (24–29). Of the limited studies that have examined HCPs' or medical students' perceptions toward the use of technology in their programs (18, 30–33), few have investigated the relationship between attitudes and performance regarding game-based assessments (34–37).

This study analyzes the survey responses of 50 HCPs to gain insight into their perceptions of the RETAIN digital training simulator for neonatal resuscitation, their attitudes toward technology, and their fixed and growth mindset. The objectives of the study are to (1) probe the validity of the survey instrument employed in this study, (2) examine whether HCPs' demographic information and previous experiences of video games and technology impact their attitudes, (3) identify different clusters of HCPs that hold similar attitudes using an unsupervised machine learning algorithm, and (4) reveal the identified clusters' long-term patterns of performance on the four tests to reveal different performance pathways through the digital simulator. The relationship between attitudinal survey responses and the game-based assessment performance is investigated to determine whether attitudes toward technology hinder or enhance HCPs' performance in a neonatal resuscitation digital simulator.

Results from the present study could inform HCPs' general conceptions of game-based simulations, technology, and mindset. Further, the revealed relationship between perceptions and performance could assist in understanding whether attitudes toward technology hinder or enhance students' learning in medical education so that instructors can appropriately incorporate new media and technology into their instruction.

## Materials and Methods

### Participants

Fifty HCPs (44 females and 6 males) were recruited from the Neonatal Intensive Care Unit (NICU) at the Royal Alexandra Hospital, Edmonton, Canada. Anonymized demographic information was collected prior to the study. The study was approved by the Human Research Ethics Board at the University of Alberta (Pro00064234). Informed consent was obtained from all HCPs prior to participation.

### Study Setup

The study was conducted based on the RETAIN (REsuscitation TrAINing for healthcare professionals) digital simulator at the simulation lab at the Centre for the Studies of Asphyxia and Resuscitation (http://retainlabsmedical.com/index.html, RETAIN Labs Medical Inc. Edmonton, Canada). RETAIN was designed for HCPs to help practice their knowledge of neonatal resuscitation through digital and tabletop simulators (37). In RETAIN, participants are asked to assume the role of an HCP and to perform interventions using the given tools, including action cards, adjustable monitors, and equipment pieces, during simulated neonatal resuscitation scenarios.

### Procedure and Data Collection

Participants completed a demographic-information questionnaire and the RETAIN digital simulator tutorial (i.e., a guided neonatal resuscitation scenario involving an apneic 24-week infant). Then, participants completed a pretest (difficult neonatal resuscitation scenario of an apneic infant with fetal bradycardia) using the RETAIN digital simulator to measure baseline performance. After completing two practice scenarios using the RETAIN digital simulator, participants repeated the assessment scenario (difficult neonatal resuscitation scenario of an apneic infant with fetal bradycardia) as a posttest to measure immediate performance change after training. Then, participants completed a questionnaire about HCPs' attitudes toward the digital simulator, technology and gaming habits, and mindset. The survey's construct validity is detailed in the Appendix (Supplementary Material). Two delayed posttests were administered to probe whether participants retained neonatal resuscitation knowledge over time. One was administered 2 months after the immediate posttest, and it was identical to the immediate posttest. The other posttest was administered 5 months after the immediate posttest (i.e., an extremely difficult scenario of an apneic infant with thick meconium) using a tabletop simulator rather than the digital simulator to examine whether HCPs transferred their neonatal resuscitation knowledge to another medium. Performance and behaviors within the digital simulator (i.e., keystrokes and mouse input automatically saved as .txt files) and tabletop simulator (i.e., ordered checklist completed by the supervising researcher) were recorded and scored.

### Measures

#### Performance Measures

The performance measures in the current study are the scores of the four game-based tests that assessed HCPs' neonatal resuscitation knowledge. Participants' performance on each assessment was scored using the 7th edition Neonatal Resuscitation Program (NRP) guidelines (38). A binary score was assigned to each performance measure: A score of one represented 100% adherence to NRP guidelines, and a score of zero represented <100% adherence to NRP guidelines.

#### Attitudinal Measures

Participants' conceptions and attitudes toward game-based assessment, technology, and their fixed and growth mindset were surveyed in the questionnaire after the immediate posttest. Growth mindset is an incremental theory of intelligence representing the belief that intelligence or ability can be improved through effort, whereas fixed mindset is an entity theory of intelligence representing the belief that intelligence or ability cannot change (39). The mindset scale includes four items using a 5-point Likert scale (1: *strongly disagree*, 2: *disagree*, 3: *neutral*, 4: *agree*, and 5: *strongly agree*). All items are positively stated except for *Fixed Mindset 1* and *2*. Reverse coding was conducted for the two items so that items on fixed mindset and growth mindset loaded on the same latent construct. All the survey questions are included in the Appendix (Supplementary Material).

#### Demographic Covariates

Participants' demographic information was collected from the questionnaire at the start of the study. It was included in the analysis to investigate whether an HCP's level of education, occupational position, and previous experience of digital games and multimedia technologies impact their attitudes toward educational games, technology in education, and mindset.

### Statistical Analyses

All analyses were performed on version 3.6.3 of the open statistical platform, *R* (40). The study is guided by the following steps.

#### Explanatory Factor Analysis

Before any analysis was conducted on the questionnaire response, an explanatory factor analysis (EFA) was performed to confirm the construct validity of the instrument. More specifically, we set out to validate that three latent factors (digital games, technology, and growth mindset) emerge from the survey responses as intended and that each item loads on the corresponding latent constructs. We used the R *psych* package to conduct the EFA analysis (41), the parallel analysis, and the very simple structure (VSS) test.

#### Partial Credit Model

Descriptive statistics from the participants' demographic information and survey response categories were reported to describe the general trend of HCPs' attitudes on the three dimensions: digital games, technology, and growth mindset. Then, a partial credit model (PCM) was employed to estimate the psychometric properties of the survey items to reveal the participants' levels of endorsement on each statement. Then, PCM with person-level covariates was fitted to examine the impact of demographic information and digital game experience on participants' attitudes. The models were fitted using the *eirm* package that enables a general linear mixed model (GLMM) analysis (conducted using the *glmer* function in the *lme4* package) (42) on a binomial dependent variable with a multilevel structure, thus overcoming the difficulty of modeling multilevel Likert scales.

#### Hierarchical Clustering

The unsupervised machine learning algorithm, agglomerative hierarchical clustering, was employed using the *hclust* function from the R *stats* package (40) to group the participants based on their survey responses. Centroids of identified clusters were reported to demonstrate the group characteristics on the three dimensions we aimed to measure. In addition, the group performances on the four tests were reported to observe the trajectories of test performance over time.

#### Generalized Linear Mixed Model

Two generalized linear mixed model (GLMM) repeated-measures analyses were conducted to track the growth of participant group performances on the four tests over time using the *glmer* function of the R *lme4* package (43).

The first GLMM model was fitted using the entire data set to test the effects of *Time* and *Group Membership* on the binary test performance (*Test score*). The within-subject repeated variable was *Time* with four levels (pretest, immediate posttest, posttest after 2 months, and posttest after 5 months), and the between-subjects variable was *Group Membership* categorized by agglomerative hierarchical clustering. The second GLMM model was fitted with data per group to identify the trajectories of performance growth within each cluster. *Time* was the within-subject repeated predictor variable. *Participant ID* was introduced as a random effect in both models.

## Results

The sample consisted of 27 registered nurses, 14 respiratory therapists, 6 doctors (including clinical fellows, residents, and clinical assistants), and 3 nurse practitioners. Participants were recruited from different educational backgrounds (diploma: 3, bachelor's degree: 24, master's degree: 4, medical degree: 6, and after degree: 4).

### Participants' Attitudes Toward the RETAIN Digital Simulator, Technology, and Mindset

**Figure 1:**
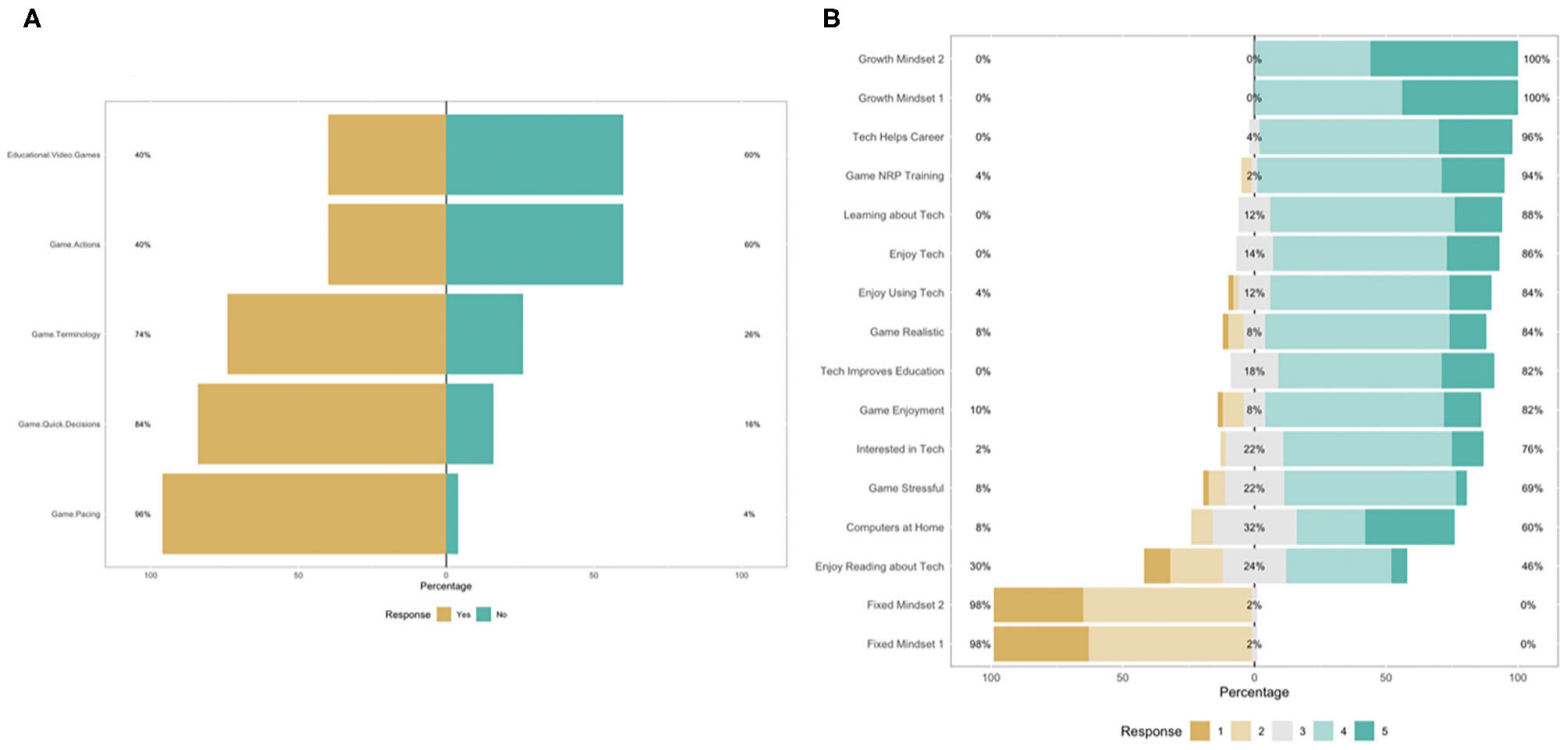
The binary **(A)** and 5-item Likert **(B)** survey items.

Figure 1 shows the survey responses on the binary questions and Likert scales. Of the 50 participants, 48 (96%) reported that the length of time and pacing during the digital simulation were appropriate to retain information of basic resuscitation steps, 37 (74%) agreed that the terminology used did not impede their ability to complete the steps, 42 (84%) reported that they could make quick decisions in the simulation-based tasks. However, 30 (60%) participants found it difficult to quickly and easily find and select the actions while playing. Participant mean years of experience in clinical neonatal care was 10.71 years (SD = 6.76), their average years of video-gaming experience was 7.46 years (SD = 9.87), and the average overall time spent on playing mobile/video games in a typical month was 6.26 (SD = 14.4). The results show that participant experience of and interest in video gaming vary widely.

Also, participants endorsed items related to *Growth Mindset* the most (100%), followed by *Technology Helps Career* (96%), *Video Game Benefits NRP Training* (94%), and *Learning about Technology* (88%). In contrast, most participants disagreed with items related to *Fixed Mindset* (98%), *Enjoy Reading about Technology* (30%), and *Computers at Home* (8%). In sum, most participants acknowledged the usefulness and potential of digital games and technology in career development, medical training, and education. Despite this, they still showed some reluctance regarding technology. Moreover, participants exhibited a high level of agreement with the growth mindset and disagreement with the fixed mindset. Participants' response categories are detailed in the Appendix (Supplementary Material).

### Construct Validity of the Survey Instrument

The parallel analysis as part of the EFA reveals that the number of factors was three. The VSS criterion determined that the VSS complexity of 1 (i.e., where all except the greatest absolute loading for an item are ignored) achieved a maximum of 0.66 with three factors, and the VSS complexity of 2 (i.e., where all except the greatest two loadings are ignored) achieved a maximum of 0.75 with three factors. Therefore, we chose the three-factor model for our data set. The factor loadings and model summaries of the EFA are presented in Table 1. Three factors were identified with sum of squared loadings (SS loadings) of 3.52, 2.74, and 1.12, respectively. All three factors are well identified as all the SS loadings are >1. Table 1 displays the factor loadings of each item on the factors identified. Thus, Items 1–4 that relate to digital simulators and game-based assessments load mainly on Factor 3. Also, Items 5–8 that relate to growth mindset load mainly on Factor 2. Last, Items 9–15 that measure attitudes toward technology load mainly on Factor 1. Thus, we assigned the following labels, Technology, Growth Mindset, and Digital Gaming, to the three factors, respectively. The correlation between Technology and Growth Mindset is 0.14 (*p* > 0.05), between Technology and Digital Gaming is 0.08 (*p* > 0.05), and between Growth Mindset and Digital Gaming is 0.07 (*p* > 0.05). The weak correlations again confirm the construct validity of our instrument as we measured diverse dimensions with the items included.

**Table 1:** EFA summary and factor loadings.

	**Item**	**Factor 1: Technology**	**Factor 2: Mindset**	**Factor 3: Digital Gaming**
**Factor loadings**				
Game Realistic	1	0.30	−0.34	0.45
Game Stressful	2	−0.05	−0.08	0.59
Game Enjoyment	3	0.26	0.13	0.33
Game Benefits NRP Training	4	0.21	0.44	0.49
Fixed Mindset 1	5	0.24	0.74	−0.24
Fixed Mindset 2	6	0.06	0.75	−0.16
Growth Mindset 1	7	−0.09	0.7	0.14
Growth Mindset 2	8	−0.14	0.74	0.25
Enjoy Reading about Technology	9	0.66	−0.31	−0.08
Enjoy Using Technology	10	0.78	0.07	−0.01
Technology Helps Career	11	0.52	0.16	0.11
Learning about Technology	12	0.63	0.01	−0.06
Interested in Technology	13	0.83	−0.1	0.07
Technology Improves Education	14	0.58	0.19	−0.04
Enjoy Using Technology to Learn	15	0.67	0.23	0.07
**Summary of explanatory factor analysis**				
Sum of Squared loadings		3.52	2.74	1.12
Proportion Variance		0.23	0.18	0.07
Cumulative Variance		0.23	0.42	0.49
Proportion Explained		0.48	0.37	0.15
Cumulative Proportion		0.48	0.85	1

### Item Psychometric Properties Estimated by the PCM

A summary of item psychometric properties from the questionnaire is presented at the top of Table 2. In accordance with results from the descriptive analysis, participant endorsement of items related to Growth Mindset 1 (Estimate = 0.668, *p* = 0.015) and Growth Mindset 2 (Estimate = 0.830, *p* = 0.004) are significantly higher than other items.

**Table 2:** Item psychometric properties estimated by the Partial Credit Model (PCM).

**Items**	**Estimates**	**SE**	***p***
Technology Helps Career	0.262	0.243	0.280
Game Enjoyment	0.111	0.236	0.638
Enjoy Reading about Technology	−0.085	0.238	0.722
Enjoy Using Technology to Learn	0.198	0.239	0.407
Enjoy Using Technology	0.111	0.236	0.638
Fixed Mindset 1	0.405	0.253	0.109
Fixed Mindset 2	0.405	0.253	0.109
Game Benefits NRP Training	0.296	0.245	0.227
Growth Mindset 1	0.668	0.275	0.015*
Growth Mindset 2	0.830	0.291	0.004**
Computers at Home	0.368	0.250	0.142
Interested in Technology	0.053	0.230	0.819
Learning about Technology	0.167	0.237	0.480
Game Realistic	0.082	0.234	0.726
Game Stressful	0.027	0.234	0.907
Technology Improves Education	0.230	0.241	0.340
**The impact of participants' demographic information and item type**			
**on their attitudes**			
Time since Last NRP Course	−0.01	0.01	0.53
Hours of Mobile/video Games at Home	0.00	0.01	0.83
Years of Video Game Experience	0.01	0.01	0.33
Experience with Educational Video Games	0.03	0.15	0.82
Education: Diploma	0.12	0.37	0.75
Education: Bachelor's	0.06	0.31	0.84
Education: After Degree	0.20	0.41	0.62
Education: Master's	0.14	0.45	0.76
Education: MD	0.17	0.24	0.48
Registration	0.12	0.17	0.46
Years of Neonatal Care Experience	−0.01	0.01	0.50

The effects of participants' demographic information and previous experience of video gaming and neonatal resuscitation on their attitudes toward technology and the RETAIN digital simulator are shown at the bottom of Table 2. Results reveal that, regardless of educational levels, occupational positions, years of neonatal care experience, and video-gaming experiences, all participants show similar attitudes in the survey with no significant differences.

### Hierarchical Clustering Based on Participants' Self-Reported Attitudes

**Figure 2:**
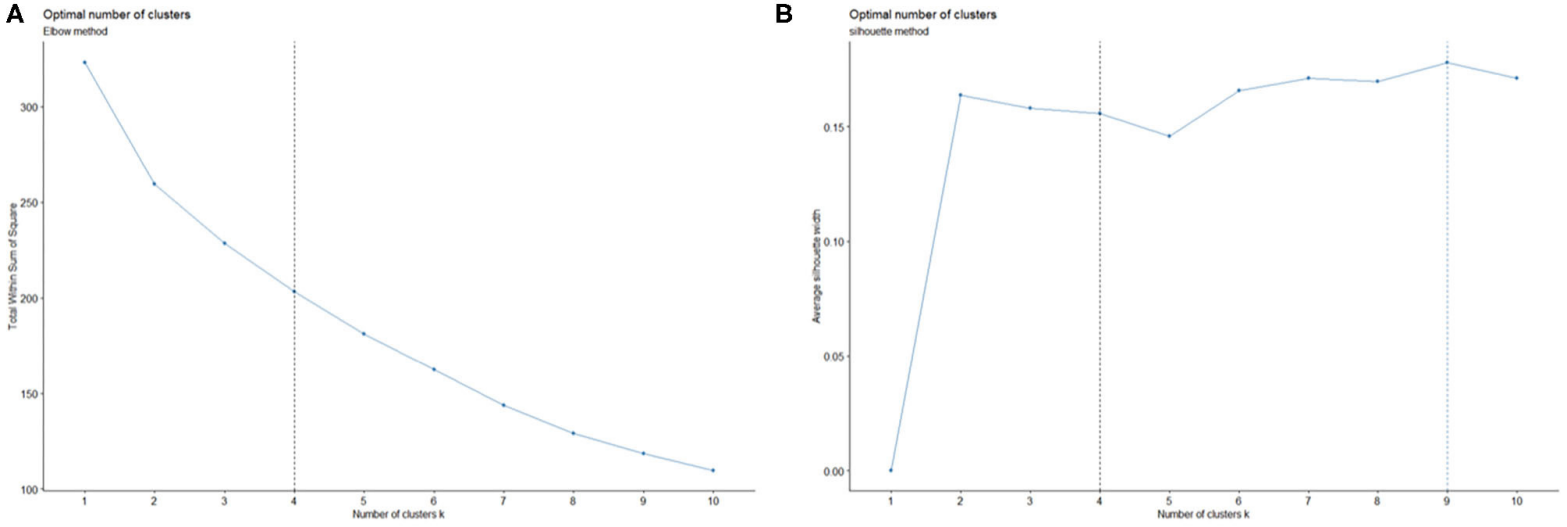
Optimal number of clusters (k) decided by the Elbow method **(A)** and the Silhouette method **(B)**.

**Figure 3:**
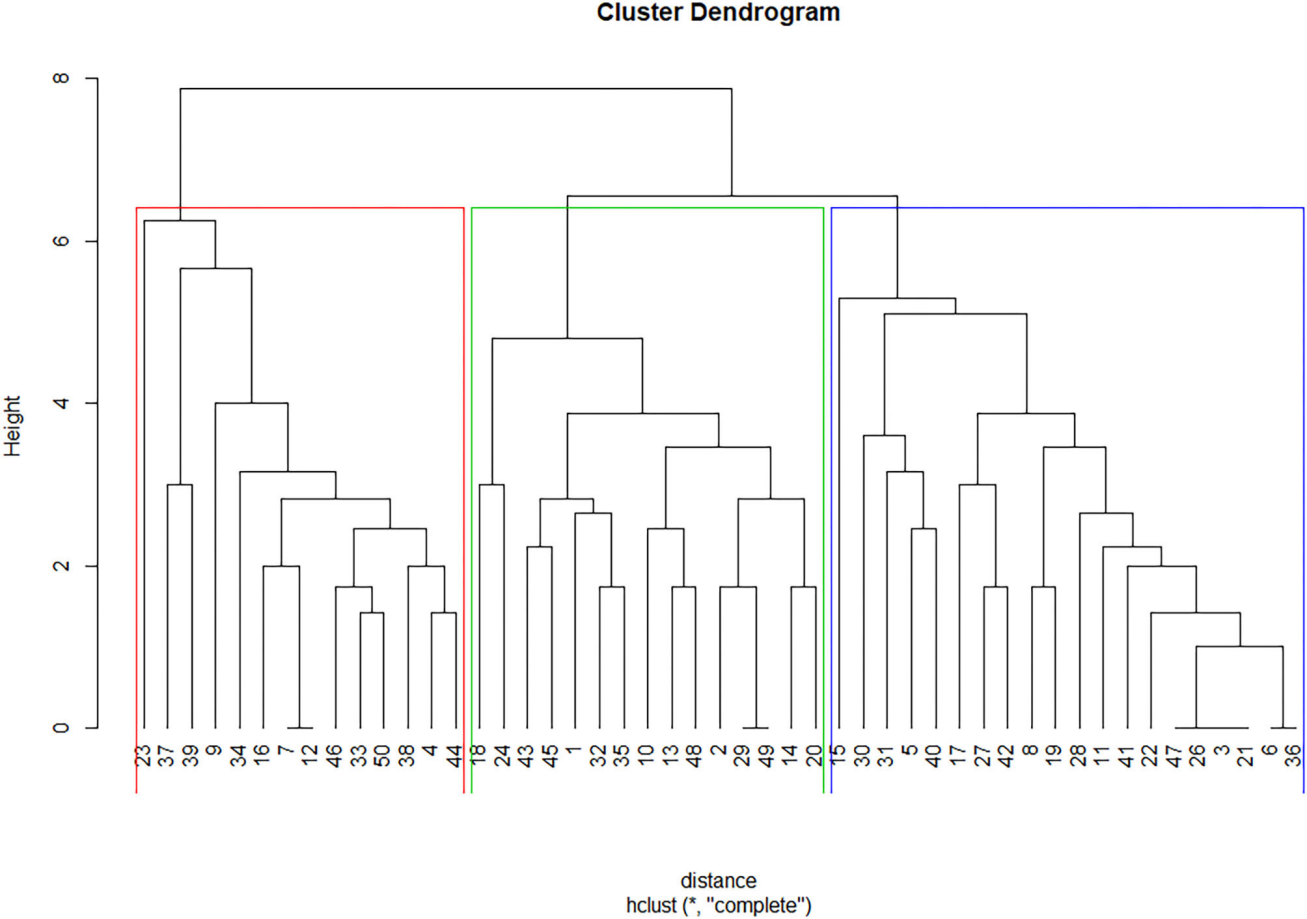
Cluster dendrogram on the cluster membership for each participant.

The unsupervised learning algorithm, hierarchical clustering, was used to cluster participants based on their survey responses. We used two methods to evaluate the optimal numbers of clusters. Both the elbow method and the silhouette method returned four as the optimal number of clusters as shown in Figure 2. However, when we set four as the number of clusters, one cluster contained only one participant, and the Euclidean distance between the cluster and the nearest cluster was too small. Therefore, we set three as the number of clusters for the present sample, which also aligns with the three-factor result of the EFA analysis. The cluster dendrogram (Figure 3) provides us with a graphical representation of the similarity in participants' attitudes and shows each participant's cluster membership and the distances between clusters.

The red circle refers to cluster 1 with 15 participants, the green circle next to cluster 1 is cluster 2 with 20 participants, and the blue circle is cluster 3 with 14 participants. One participant did not complete the survey and, thus, was excluded from the clustering analysis. To further explore attitude differences among clusters, we extracted the centroids (i.e., the middle or the average of a cluster) of each cluster to represent their characteristics on the dimensions we measured as shown at the top of Table 3. The cluster dendrogram and details regarding this method can be found in the Appendix (Supplementary Material).

**Table 3:** Centroids of the three clusters on their survey responses.

		**Cluster 1**			**Cluster 2**			**Cluster 3**	
**Attitude survey items responses**									
N of participants		15			20			14	
Game Realistic		4.33			3.70			3.64	
Game Stressful		3.47			3.60			3.86	
Game Enjoyment		4.20			3.40			4.14	
Game Benefits NRP Training		4.20			3.90			4.36	
Fixed Mindset 1		4.47			4.15			4.43	
Fixed Mindset 2		4.40			4.10			4.50	
Growth Mindset 1		4.53			4.20			4.64	
Growth Mindset 2		4.67			4.25			4.86	
Enjoy Reading about Technology		4.13			3.25			2.00	
Enjoy Using Technology		4.47			3.70			3.70	
Enjoy Technology		4.47			3.90			3.79	
Technology Helps Career		4.53			4.00			4.36	
Learning about Technology		4.47			3.90			3.93	
Technology Interest		4.40			3.75			3.50	
Technology in Education		4.40			4.00			3.57	
	**Cluster 1**			**Cluster 2**			**Cluster 3**		
	***M***		***SD***	***M***		***SD***	***M***		***SD***
**Three clusters' mean performance scores on the four consecutive tests with standard deviations**									
Pretest	0.46		0.52	0.44		0.40	0.36		0.51
Posttest_Immediate	0.77		0.51	0.81		0.47	0.73		0.38
Posttest_2month	0.77		0.51	0.69		0.44	0.63		0.48
Posttest_5month	0.85		0.44	0.69		0.48	0.91		0.30
	**Est**	**SE**	***p***	**Est**	**SE**	***p***	**Est**	**SE**	***p***
**General linear mixed modeling**									
(Intercept)	−0.41	0.53	0.44	−0.84	0.59	0.16	−0.19	0.64	0.77
Time2_Immediate Posttest	1.79	0.83	0.03	2.31	0.78	0.00	1.64	0.92	0.07
Time3_Posttest after 2 months	1.61	0.84	0.06	1.86	0.79	0.02	0.65	0.85	0.44
Time4_Posttest after 5 months	2.11	0.93	0.02	1.76	0.80	0.03	2.93	1.30	0.02
AIC		72.4			97.1			65.8	
BIC		86.4			110.8			77.2	
Log Likelihood		−30.2			−42.5			−26.9	
Deviance		60.4			85.1			53.8	
Residual degrees of freedom		50			67			43	

*M, Mean; SD, Standard Deviation; Est, Estimate; SE, Standard Error*.

Cluster 1 shows the highest endorsement on most items in the survey compared with the other two clusters, and it shows positive attitudes toward the digital simulator, strong endorsement of growth mindset, and great interest and openness to new technology. Cluster 2 shows neutral attitudes on most items. Cluster 3 shows the highest levels of agreement on items related to growth mindset and *Game Benefits NRP Training*. Although cluster 3 endorses the highest growth mindset and supports the idea of technology helping careers, it shows the least endorsement of using new technology.

### Cluster Performance on the Four Summative Assessments

We fitted two GLMMs: The first model probed the effects of *Time* as a within-subject factor and of *Cluster Membership* as a between-subjects factor on participants' test performance. The second model fit the data from different clusters with only *Time* as a within-subject repeated measure to observe each cluster's long-term performance trajectories.

**Figure 4:**
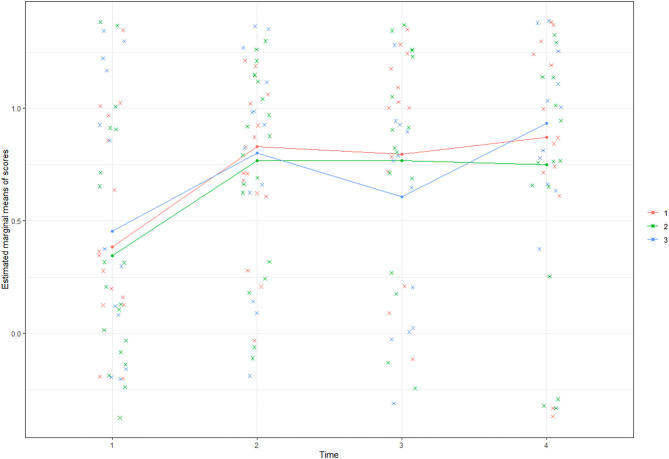
The estimated marginal means of performance scores by clusters.

The first model [summarized in the Appendix (Supplementary Material)] reveals that participants performed significantly better on all posttests (Estimate_immediate_ = 2.05, *p* < 0.02; Estimate_2−month_ = 1.84, *p* < 0.05; Estimate_5−month_ = 2.38, *p* < 0.05) compared with their performance on the pretest. However, there are no significant performance differences among the three clusters. The performance growth trajectories per cluster (Figure 4) are estimated by the marginal means. The results show that, after the simulation-based training for neonatal resuscitation, participants from the three clusters all showed knowledge gains in the transfer test (the posttest after 5 months) regardless of their attitudinal pathways.

Results of the GLMM for the three clusters can be found at the bottom of Table 3, which shows that cluster 1 participants' performance was significantly better on the immediate posttest compared with the pretest (Estimate = 1.79, *p* = 0.03) but slightly decreased after 2 months (with no significant difference from the pretest) and increased the most on the last posttest (Estimate = 2.11, *p* = 0.02), which was delayed 5 months. Cluster 2 participants scored significantly higher on the immediate posttest compared with the pretest, and their performance declined slightly on the following tests. However, on the last transfer test, their scores were still significantly higher than the pretest scores (Estimate = 1.76, *p* = 0.03). Importantly, cluster 3 scored the lowest on the pretest (*M* = 0.36) and the highest on the last posttest (*M* = 0.91). Moreover, participants from cluster 3 did not achieve a significantly higher performance compared to the pretest until the last transfer test. This indicates that the tabletop simulator picks up participants' knowledge that may not have been totally captured by the tests in the digital simulator.

## Discussion

Simulation-based neonatal resuscitation training and game-based assessments can provide HCPs with more opportunities to practice their procedural knowledge with real-life scenarios in a low-stakes environment (7). Few studies have examined HCPs' attitudes toward digital simulations, technology, and mindset and even fewer have linked HCPs' attitudes toward knowledge acquisition, retention, and transfer. This study examines 50 HCPs' perceptions of a digital simulator, their attitudes toward technology, and also their self-reported theories of intelligence (i.e., mindsets). Results show that most participants agree that technology and digital simulators can benefit neonatal resuscitation training and career development regardless of their levels of education, positions, years of neonatal care experience, and video-game experience. Moreover, all participants demonstrate strong endorsement of a growth mindset.

In addition, we used unsupervised machine learning to cluster participants based on their attitudes. Three clusters were identified: high endorsement on most items (cluster 1), neutral attitudes toward all survey items (cluster 2), and the highest levels of agreement with growth mindset concomitant with the lowest interest and enjoyment regarding technology despite their acknowledgments of the benefits of digital simulators for neonatal resuscitation and of technology for career development (cluster 3).

To further examine the three clusters' performance growth and knowledge acquisition trajectories, we fitted the data into a GLMM to observe whether the participants learned and retained the neonatal resuscitation knowledge after the simulation-based training program. Results reveal that, although the three clusters underwent disparate attitudinal paths, all participants learned, retained, and transferred neonatal resuscitation knowledge after the digital simulator intervention. More specifically, participants from cluster 1 with high motivation and interest in games and technology showed significant performance progress on the immediate posttest, and although their scores slightly declined on the first delayed posttest, they improved their performance again on the transfer test. Cluster 2 shows a neutral attitude on most survey items compared with the other two clusters. Similar to cluster 1, participants from cluster 2 improved their performance significantly on the immediate posttest. However, they failed to improve more on the following tests. Last, cluster 3 started with the lowest scores among the three groups and did not improve their performance significantly on the immediate posttest or on the test after 2 months, which were both administered using the digital simulator. However, they improved the performance significantly on the posttest after 5 months that was administered using a tabletop simulator, achieving the highest performance on the last posttest that measured knowledge transfer compared with the other clusters.

Although all three clusters show that participants improved their performance over time and transferred their knowledge to a new medium (from digital to traditional board games), the results for cluster 3 show that technology is not a prerequisite for performance gain from a digital simulator and that there may be different pathways to achieving better performance as these participants were able to gain the most from the pretest to the last posttest. The low interest in and enjoyment of technology may have weakened their performances on the computer-based assessments. However, the intervention from the digital simulation–based training helped the participants learn and retain knowledge. They were able to greatly improve their performance when the assessment medium was changed even when the assessment content was more difficult than in previous tests. Although cluster 3 participants were less likely to enjoy technology or digital simulators, they still benefited from using the technology-based digital simulation and transferred their knowledge on the tabletop simulator perhaps due to their high endorsement of a growth mindset. Moreover, an individual's growth mindset could be a pathway to achieving high performance on neonatal resuscitation, especially when learners do not embrace technology; therefore, mindset interventions could be useful in this domain. Negative attitudes toward digital simulators and technology may hinder participant performance in the short term, but their growth mindset may help them learn and transfer their knowledge in the long term. Overall, this study suggests that novel simulators (digital or tabletop) may be a viable alternative to traditional training in neonatal resuscitation.

## Conclusion

With the pervasiveness of multimedia and technology, it is important to examine individuals' attitudes and understand how digital simulators could promote neonatal resuscitation training with lower costs and risks. Results show that, although most participants acknowledge the benefits of digital simulators and technology in neonatal resuscitation training, they are reluctant to embrace technology. Three clusters were identified based on participants' attitudes toward digital simulators technology, and mindset. Further, participants have learned, retained, and transferred neonatal resuscitation knowledge over time even if they followed different learning paths determined by their attitudes. Results indicate the potential of digital simulators in training and assessing HCPs that can capture performance expressed through different learning paths.

## Data Availability Statement

All datasets generated for this study are included in the article/Supplementary Material.

## Ethics Statement

The studies involving human participants were reviewed and approved by the Human Research Ethics Board at the University of Alberta (Pro00064234). Informed consent was obtained from all HCPs prior to participation. The patients/participants provided their written informed consent to participate in this study.

## Author Contributions

GS, SG, MC, and CL conceptualized and designed the study, drafted the initial manuscript, and critically reviewed and revised the manuscript. All authors approved the final manuscript as submitted and agree to be accountable for all aspects of the work.

## Conflict of Interest

GS is an owner of RETAINLabs Medical Inc., Edmonton, Canada (http://retainlabsmedical.com), which is distributing the game. GS has registered the RETAIN table-top simulator (Tech ID 2017083) and the RETAIN digital simulator under Canadian copyright (Tech ID—2017086). The remaining authors declare that the research was conducted in the absence of any commercial or financial relationships that could be construed as a potential conflict of interest.

## Abbreviations

HCP: Healthcare professional
NICU: Neonatal Intensive Care Unit
NRP: Neonatal Resuscitation Program
RETAIN: REsuscitation TrAINing for healthcare professionals
SD: Standard Deviation
SE: Standard Error
Est: estimate
M: mean
GLMM: generalized linear mixed model
PCM: partial credit model
VSS: very simple structure
EFA: explanatory factor analysis.
