# Supplementary material for: Unsupervised Machine Learning Algorithms Examine Healthcare Providers' Perceptions and Longitudinal Performance in a Digital Neonatal Resuscitation Simulator
Source: Front Pediatr. 2020 Sep 11;8:544. doi: 10.3389/fped.2020.00544 (PMC7518390; doi:10.3389/fped.2020.00544)
Supplement: Supplementary file 2 [file Data_Sheet_2.docx]

Supplementary Material

# The Distribution of the Survey Responses

Table A1. Summary of participants’ response category on the 5-point Likert scale.

|  | Strongly Disagree | Disagree | Neutral | Agree | Strongly Agree | Non-response |
| --- | --- | --- | --- | --- | --- | --- |
| Game Realistic | 1 | 3 | 4 | 35 | 7 | 0 |
| Game Stressful | 1 | 3 | 11 | 32 | 2 | 1 |
| Game Enjoyment | 1 | 4 | 4 | 34 | 7 | 0 |
| Game Benefits NRP Training | 0 | 2 | 1 | 35 | 12 | 0 |
| Fixed Mindset1 | 0 | 0 | 1 | 31 | 18 | 0 |
| Fixed Mindset2 | 0 | 0 | 1 | 32 | 17 | 0 |
| Growth Mindset1 | 0 | 0 | 0 | 28 | 22 | 0 |
| Growth Mindset2 | 0 | 0 | 0 | 22 | 28 | 0 |
| Enjoy Reading with Technology | 5 | 10 | 12 | 20 | 3 | 0 |
| Enjoy Using Technology | 1 | 1 | 6 | 34 | 8 | 0 |
| Technology Helps Career | 0 | 0 | 2 | 34 | 14 | 0 |
| Learning with Technology | 0 | 0 | 6 | 35 | 9 | 0 |
| Technology Interest | 0 | 1 | 11 | 32 | 6 | 0 |
| Technology in Education | 0 | 0 | 9 | 31 | 10 | 0 |
| Enjoy Technology | 0 | 0 | 7 | 33 | 10 | 0 |

# GLMM: Model 1

Performance of Cluster 1 started the highest on the pre-test, increased significantly on the immediate post-test, then decreased on the 2-month delayed test within the digital simulator, and, finally, increased again on the 5-month delayed test within the table-top simulator. Cluster 2 started with low scores, increased significantly on the immediate post-test, and kept decreasing on subsequent tests. Cluster 3 started with low scores, increased on the immediate post-test, then decreased greatly on the 2-month delayed post-test.

Table A2. GLMM with the within-subject variable *Time* and the between-subject variable *Cluster Membership*.

|  | | *M* | | | *SD* | | |
| --- | --- | --- | --- | --- | --- | --- | --- |
| Pre-test | | 0.42 | | | 0.5 | | |
| Post-test_Immediate | | 0.78 | | | 0.42 | | |
| Post-test_2month | | 0.70 | | | 0.46 | | |
| Post-test_5month | | 0.80 | | | 0.41 | | |
| *General Linear Mixed Model* | | | | | | | |
|  | | Estimate | | SE | | | *p* |
| (Intercept) | | -0.47 | | 0.61 | | | .44 |
| Time2_Immediate Post-test | | **2.05** | | **0.91** | | | **.02** |
| Time3_Post-test after 2 months | | **1.84** | | **0.92** | | | **.05** |
| Time4_Post-test after 5 months | | **2.38** | | **1.01** | | | **.02** |
| cluster2 | | -0.16 | | 0.82 | | | .84 |
| cluster3 | | 0.29 | | 0.89 | | | .74 |
| Time2:cluster2 | | -0.21 | | 1.17 | | | .85 |
| Time3:cluster2 | | -0.01 | | 1.21 | | | 1.00 |
| Time4:cluster2 | | -0.65 | | 1.28 | | | .61 |
| Time2:cluster3 | | -0.48 | | 1.28 | | | .71 |
| Time3:cluster3 | | -1.22 | | 1.26 | | | .33 |
| Time4:cluster3 | | 0.44 | | 1.59 | | | .78 |
| AIC | | 222.9 | | | | | |
| BIC | | 267.1 | | | | | |
| Log likelihood | | -97.5 | | | | | |
| Deviance | | 194.9 | | | | | |
| Degrees of freedom residual | | 160 | | | | | |
| *Pairwise comparisons on the performance measures between the clusters* | | | | | | | |
| Contrast | Estimate | | SE | | | *p* | |
| 1-2 | **-1.82** | | 0.53 | | | .00 | |
| 1-3 | **-1.43** | | 0.52 | | | .03 | |
| 1-4 | **-2.31** | | 0.63 | | | .00 | |
| 2-3 | 0.40 | | 0.53 | | | .88 | |
| 2-4 | -0.49 | | 0.62 | | | .86 | |
| 3-4 | -0.89 | | 0.62 | | | .49 | |

Note: M: Mean, SD: Standard Deviation, SE = Standard Error.
