# Supplementary material for: Unsupervised Machine Learning Algorithms Examine Healthcare Providers' Perceptions and Longitudinal Performance in a Digital Neonatal Resuscitation Simulator
Source: Front Pediatr. 2020 Sep 11;8:544. doi: 10.3389/fped.2020.00544 (PMC7518390; doi:10.3389/fped.2020.00544)
Supplement: Supplementary file 3 [file Data_Sheet_3.docx]

**Survey Instrument**

**Pre-Survey**

Which best describes your gender identity?

1. Female
2. Male
3. Other

Time (in months) since your last NRP course:

What is the highest level of education you have completed?

1. Diploma, certificate, or other professional program
2. Bachelor’s degree (e.g., B.Sc.N.)
3. After degree
4. Master’s degree
5. MD
6. PhD
7. Other:

What is your registration(s), if any?

1. MD – Neonatologist
2. NNP
3. MD – Fellow / Resident
4. RN
5. RT
6. Other:

What is your current position?

1. Physician
2. NNP / Fellow / Resident
3. Nurse
4. Respiratory Therapist
5. Student nurse / Medical student
6. Other:

How many years of experience in clinical neonatal care do you have?

**Post-Survey**

The length of time and pacing during the game was appropriate to retain information of basic resuscitation steps.

1. Yes
2. No

The terminology used did not impede your ability to complete the steps (i.e. the terminology was appropriate).

1. Yes
2. No

You could make decisions quickly.

1. Yes
2. No

The actions you wished to perform were available, and you could quickly and easily find and select them while playing.

1. Yes
2. No

The scenario was realistic.

1. Yes
2. No

The scenario simulated the stressful nature of neonatal resuscitation.

1. Strongly disagree
2. Disagree
3. Neutral
4. Agree
5. Strongly agree

Did you enjoy playing this game?

1. Strongly disagree
2. Disagree
3. Neutral
4. Agree
5. Strongly agree

What did you like about the game?

What did you dislike about the game?

Other comments/changes/improvements about the game?

Do you believe that this game could be beneficial for NRP training?

1. Strongly disagree
2. Disagree
3. Neutral
4. Agree
5. Strongly agree

You can’t really do much to change how good you are at your job.

1. Strongly disagree
2. Disagree
3. Neutral
4. Agree
5. Strongly agree

You can learn new things, but you cannot really change how good you are at your job.

1. Strongly disagree
2. Disagree
3. Neutral
4. Agree
5. Strongly agree

You can always change how good you are at your job.

1. Strongly disagree
2. Disagree
3. Neutral
4. Agree
5. Strongly agree

You can get better at your job with practice.

1. Strongly disagree
2. Disagree
3. Neutral
4. Agree
5. Strongly agree

How many hours do you spend playing mobile/video games in a typical month?

How many overall years of video gaming experience do you have?

Do you have any previous experience with educational video games?

1. Yes
2. No

I enjoy reading about technology.

1. Strongly disagree
2. Disagree
3. Neutral
4. Agree
5. Strongly agree

I enjoy using technology.

1. Strongly disagree
2. Disagree
3. Neutral
4. Agree
5. Strongly agree

I believe technology can be used in education to improve learning.

1. Strongly disagree
2. Disagree
3. Neutral
4. Agree
5. Strongly agree

Making an effort to learn new technologies will help me in my career.

1. Strongly disagree
2. Disagree
3. Neutral
4. Agree
5. Strongly agree

I look forward to learning new technologies.

1. Strongly disagree
2. Disagree
3. Neutral
4. Agree
5. Strongly agree

I am interested in the things I learn about technology.

1. Strongly disagree
2. Disagree
3. Neutral
4. Agree
5. Strongly agree

I enjoy using technology to help me learn new things.

1. Strongly disagree
2. Disagree
3. Neutral
4. Agree
5. Strongly agree

How many hours do you spend using smartphones/computers/laptops/tablets at home each day?

1. I don’t use a computer
2. Less than 1 hour
3. 1-2 hours
4. 2-3 hours
5. 3 or more hours
